# Consensus computational network analysis for identifying candidate outer membrane proteins from *Borrelia* spirochetes

**DOI:** 10.1186/s12866-016-0762-z

**Published:** 2016-07-11

**Authors:** Melisha R. Kenedy, Edgar J. Scott, Binu Shrestha, Arvind Anand, Henna Iqbal, Justin D. Radolf, David W. Dyer, Darrin R. Akins

**Affiliations:** Department of Microbiology and Immunology, University of Oklahoma Health Sciences Center, Oklahoma City, Oklahoma 73104 USA; Department of Medicine, University of Connecticut Health Center, Farmington, Connecticut 06030 USA; Department of Pediatrics, University of Connecticut Health Center, Farmington, Connecticut 06030 USA; Department of Genetics and Genomic Science, University of Connecticut Health Center, Farmington, Connecticut 06030 USA; Department of Immunology, University of Connecticut Health Center, Farmington, Connecticut 06030 USA; Department of Molecular Biology and Biophysics, University of Connecticut Health Center, Farmington, Connecticut 06030 USA

**Keywords:** *Borrelia*, Lyme disease, Relapsing fever, Outer membrane proteins

## Abstract

**Background:**

Similar to Gram-negative organisms, *Borrelia* spirochetes are dual-membrane organisms with both an inner and outer membrane. Although the outer membrane contains integral membrane proteins, few of the borrelial outer membrane proteins (OMPs) have been identified and characterized to date. Therefore, we utilized a consensus computational network analysis to identify novel borrelial OMPs.

**Results:**

Using a series of computer-based algorithms, we selected all protein-encoding sequences predicted to be OM-localized and/or to form β-barrels in the borrelial OM. Using this system, we identified 41 potential OMPs from *B. burgdorferi* and characterized three (BB0838, BB0405, and BB0406) to confirm that our computer-based methodology did, in fact, identify borrelial OMPs. Triton X-114 phase partitioning revealed that BB0838 is found in the detergent phase, which would be expected of a membrane protein. Proteolysis assays indicate that BB0838 is partially sensitive to both proteinase K and trypsin, further indicating that BB0838 is surface-exposed. Consistent with a prior study, we also confirmed that BB0405 is surface-exposed and associates with the borrelial OM. Furthermore, we have shown that BB0406, the product of a co-transcribed downstream gene, also encodes a novel, previously uncharacterized borrelial OMP. Interestingly, while BB0406 has several physicochemical properties consistent with it being an OMP, it was found to be resistant to surface proteolysis. Consistent with BB0405 and BB0406 being OMPs, both were found to be capable of incorporating into liposomes and exhibit pore-forming activity, suggesting that both proteins are porins. Lastly, we expanded our computational analysis to identify OMPs from other borrelial organisms, including both Lyme disease and relapsing fever spirochetes.

**Conclusions:**

Using a consensus computer algorithm, we generated a list of candidate OMPs for both Lyme disease and relapsing fever spirochetes and determined that three of the predicted *B. burgdorferi* proteins identified were indeed novel borrelial OMPs. The combined studies have identified putative spirochetal OMPs that can now be examined for their roles in virulence, physiology, and disease pathogenesis. Importantly, the studies described in this report provide a framework by which OMPs from any human pathogen with a diderm ultrastructure could be cataloged to identify novel virulence factors and vaccine candidates.

**Electronic supplementary material:**

The online version of this article (doi:10.1186/s12866-016-0762-z) contains supplementary material, which is available to authorized users.

## Background

Pathogenic spirochetes belonging to the genus *Borrelia* cause Lyme disease and relapsing fever, both of which are vector-borne illnesses. Lyme disease is caused by pathogenic spirochetes of the *Borrelia burgdorferi* sensu lato complex which are transmitted to humans through the bite of hard-bodied *Ixodes* ticks [1, 2]. The earliest manifestations of Lyme disease include a characteristic skin rash, termed erythema migrans, along with concomitant flu-like symptoms followed by disorders of the heart, nervous system, and joints [1]. Globally, most cases of Lyme disease can be attributed to three *Borrelia* genospecies, *B. burgdorferi* sensu stricto (hereafter referred to as *B. burgdorferi*), *B. afzelii*, and *B. garinii*. In recent years, however, the number of genospecies associated with Lyme disease has expanded to include other organisms such as *B. bissettii*, *B. valaisiana*, *B. spielmanii*, and *B. lusitaniae* [3–7]. A second group of *Borrelia* spirochetes including *B. hermsii*, *B. recurrentis*, *B. duttonii*, *B. parkeri*, *B. crocidurae*, *B. miyamotoi*, and *B. turicatae*, are the causative agents of relapsing fever, a disease characterized by recurring episodes of fever with muscle and joint aches [8, 9]. Relapsing fever *Borrelia* are generally transmitted to humans by a soft-bodied tick of the genus *Ornithodoros*; however, one genospecies, *B. recurrentis*, is transmitted by the body louse [2].

Similar to Gram-negative organisms, *Borrelia* spirochetes are dual-membrane organisms with both an inner membrane and an outer membrane (OM); however, *Borrelia* species lack lipopolysaccharide [10, 11]. Instead, the surface of *Borrelia* spirochetes is characterized by the presence of numerous surface-exposed lipoproteins that are attached to the outer leaflet of the OM via N-terminal lipid moieties [12]. Borrelial lipoproteins have been the focus of intense study for several decades and are known to be important in virulence and host-pathogen interactions [11, 13–23]. Many of the borrelial lipoproteins are plasmid-encoded and differentially expressed throughout the life cycle of the organism [23–28].

The OM of Gram-negative organisms contain membrane-spanning, outer membrane proteins (OMPs) that form amphipathic β-barrels that can typically form nonspecific or substrate-specific OM pores [29, 30]. Freeze-fracture electron microscopy of the *B. burgdorferi* OM confirmed that the borrelial OM also possess integral OMPs, although the number of OMPs in the borrelial OM is at least 10-fold reduced as compared to *Escherichia coli* [31, 32]. The *Borrelia* OMPs identified to date are implicated in nutrient acquisition, antibiotic resistance, host-pathogen interactions, protein transport and assembly, and pore formation [33–42]. These proteins are characterized by their OM-localization and/or surface exposure but are not lipid-modified lipoproteins. Unlike the majority of the surface-exposed lipoproteins, all of the borrelial OMPs identified thus far are encoded on the ~900 kB linear chromosome [11, 33, 39, 41, 43–46]. While it is known that the borrelial OM contains membrane spanning OMPs with β-barrel structure and/or pore-forming capabilities that are important in overall physiology and host interactions, fewer than 10 borrelial proteins have been identified as potential OMPs and only half of those have thus far been characterized [22, 33, 39, 41, 45–52]. Of the known borrelial OMPs, *B. burgdorferi* proteins BamA (BB0795), BesC (BB0142), DipA (BB0418), P66 (BB0603), and P13 (BB0034) have been shown to form a β-barrel, to form pores in the borrelial OM, or to be functional orthologs to known OMPs. Efforts to identify novel OMPs in *Borrelia* spirochetes have been hindered for several reasons, including the low abundance of borrelial OMPs in the *Borrelia* OM and the unique fragility of the borrelial OM. Moreover, few orthologs to well-characterized proteins from other bacterial organisms have been identified through sequence comparison analyses [11, 20].

Given the challenges of identifying borrelial OMPs using conventional biochemical and proteomic approaches, we created a bioinformatics approach that utilizes an algorithm for predicting OMPs based on their unique properties and secondary structures. Using the derived computer-based algorithm, we examined chromosomally encoded proteins from both Lyme disease and relapsing fever *Borrelia* to identify candidate proteins that were both conserved and predicted to be OMPs in the genus *Borrelia*. Known borrelial OMPs were identified on the final candidate OMP list; and, importantly, three of the previously uncharacterized *B. burgdorferi* proteins identified by this method (BB0838, BB0405, and BB0406) were confirmed to be OMPs, indicating the computer-based methodology could, in fact, predict novel borrelial OMPs. Specifically, we determined that the *B. burgdorferi* protein BB0838 is amphiphilic and has surface-exposed regions that were accessible to proteases. *B. burgdorferi* BB0405 was previously shown by our laboratory to be a surface-exposed protein that localized to the OM [22, 39], and BB0405 and BB0406 were detected in OM vesicles by Pal and colleagues in a study examining the overall protein and lipoprotein content of the borrelial OM [53]. Herein, we found that BB0406 was indeed amphiphilic and localized to the *B. burgdorferi* OM as shown previously for BB0405 and that both BB0405 and BB0406 are pore-forming proteins.

## Results

### Computational framework for predicting *Borrelia burgdorferi* B31 outer membrane proteins

**Fig. 1:**
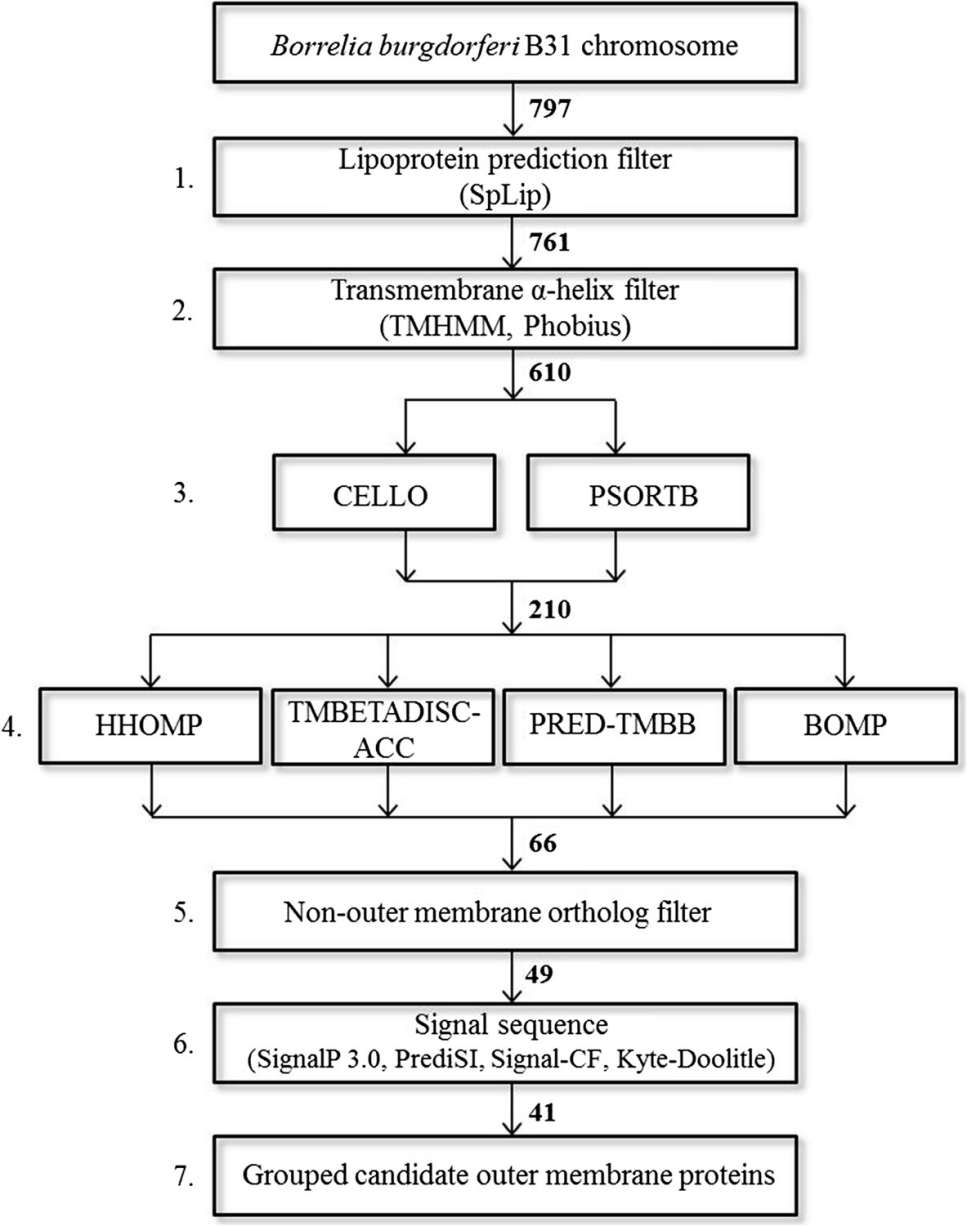
Computational framework for predicting OM-localized, β-barrel proteins from *B. burgdorferi* B31. All proteins encoded on the *B. burgdorferi* B31 chromosome were subjected to filters as shown. The following steps outline the computational framework utilized in the study: (1) proteins predicted to be spirochaetal lipoproteins were discarded, (2) proteins predicted to contain transmembrane α-helices by one of two programs were discarded, (3) proteins predicted to be OM-localized by one of two programs were retained, (4) proteins predicted to form a β-barrel by at least one of four or two of four β-barrel prediction programs depending on if the sequences was predicted to be OM-localized by two or one of the OM-localization programs were retained, (5) proteins orthologous to known non-OMPs were discarded, (6) proteins predicted to have a N-terminal signal peptide by at least one of four programs were retained, and (7) the remaining proteins were categorized by the number of programs predicting OM-localization or propensity to form a β-barrel. The number of sequences remaining after each filter are indicated. Proteins were removed from the candidate OMP list if the sequence was not predicted to be OM localized and have β-barrel conformation by three of the following six algorithims: CELLO, pSORTb, HHOMP, TMBETADISC-AAC, PRED-TMBB, and BOMP

To date, few *B. burgdorferi* outer membrane proteins (OMPs) have been identified [22, 33, 39, 41, 50]; therefore, we aimed to identify novel *B. burgdorferi* B31 OMPs that are localized to the OM but are not borrelial lipoproteins. We used a bioinformatics strategy (outlined in Fig. 1) modified from one used recently to predict OMPs in the spirochete *Treponema pallidum* [54]. Given that all *Borrelia* OMPs identified to date are chromosomally encoded and that we anticipate chromosomally encoded proteins will be more conserved among *Borrelia* strains [33, 39, 41, 46, 50], we focused on identifying novel OMPs encoded on the *B. burgdorferi* B31 chromosome. As summarized in Fig. 1, to generate a list of candidate *B. burgdorferi* OMPs, we eliminated predicted lipoproteins and proteins predicted to contain transmembrane α-helices, retained protein sequences predicted to be OM-localized and/or to form β-barrels, removed sequences that were orthologous to known non-OMPs, and retained proteins with predicted N-terminal signal peptides. More specifically, all protein sequences encoded from the *B. burgdorferi* B31 chromosome were first analyzed for their potential to be a spirochaetal lipoprotein according to the methods described by Setubal, et al. [55] and/or to contain transmembrane α-helices by either one of two algorithms [Phobius [56] and TMHMM [57]]. As neither of these properties are characteristic of integral OMPs, all sequences predicted to encode membrane anchored lipoproteins or to contain one or more alpha-helical transmembrane domains were eliminated from the candidate OMP list. The 610 sequences remaining were then examined for their likelihood to encode a protein localized to the OM and predicted to form a β-barrel. Any sequence predicted to be OM-localized by one of two programs that predict cellular localization [CELLO [58] and/or pSORTb 3.0 [59]] was retained as a candidate OMP and was then examined for β-barrel topology given that all structurally characterized OMPs from diderm organisms form β-barrels. The programs HHOMP [60], TMBDISC-ACC [61], PRED-TMBB [62], and BOMP [63] were used to examine β-barrel propensity, and proteins were retained if they were predicted to be a β-barrel by at least one of the four or two of the four β-barrel prediction programs depending on if the sequence was predicted to be OM-localized by two or one of the OM-localization programs, respectively. Next, any sequence orthologous to proteins that are known not to be OMPs in other organisms were excluded from the list of candidate borrelial OMPs. Finally, we also analyzed the first 60 amino acids of the remaining sequences for the presence of a canonical N-terminal signal peptide. Given that proteins localized to the OM would require a signal peptide, we removed from the list any protein not predicted to have a signal peptide by at least one of four signal peptide prediction programs [64–67]. A final list of 41 *B. burgdorferi* B31 candidate OMPs was compiled (Table 1). To further prioritize this list, we categorized sequences according to the number of computational programs predicting OM localization and/or β-barrel topology from the following six programs mentioned above: CELLO, pSORTb 3.0, HHOMP, TMBDISC-ACC, PRED-TMBB, and BOMP. For instance, our highest priority candidate OMPs are those sequences for which all six programs predicted the encoded protein to be an OMP, and we considered any that were predicted by three or more of the localization and/or β-barrel topology programs to be putative OMPs worthy of further analysis.

**Table 1:** *Borrelia burgdorferi* B31 candidate OMPs

Protein	Outer membrane localization/β-barrel conformation^*a*^						Signal sequence^*b*^			
	CELLO	PSORTb	HHOMP	TMBETADISC -ACC	PRED-TMBB	BOMP	SignalP 3.0	PrediSI	Signal-CF	Kyte Doolittle
Group 1 (6/6)										
BB0418 (DipA)	+	+	+	+	+	+	+	+	+	+
BB0794	+	+	+	+	+	+	-	+	+	+
BB0795 (BamA)	+	+	+	+	+	+	-	+	+	+
BB0838	+	+	+	+	+	+	+	+	+	+
Group 2 (5/6)										
BB0110	+	+	+	+	+	-	-	-	-	+
BB0236	+	+	-	+	+	+	-	+	-	+
BB0603 (P66)	+	+	+	+	+	-	+	+	+	+
BB0667	+	+	-	+	+	+	-	+	-	+
BB0811	+	+	-	+	+	+	+	+	+	+
BB0824	+	-	+	+	+	+	-	+	+	+
Group 3 (4/6)										
BB0027	+	-	+	+	+	-	-	-	+	+
BB0089	+	+	-	+	-	+	+	+	+	+
BB0142 (BesC)	+	-	+	+	+	-	-	-	-	+
BB0156	+	+	-	+	+	-	-	-	+	+
BB0159	+	+	-	+	+	-	+	-	-	+
BB0161	+	+	+	-	-	+	-	-	-	+
BB0308	+	+	-	+	-	+	-	+	+	+
BB0319	+	+	-	+	-	+	-	-	-	+
BB0464	+	+	-	+	-	+	+	+	+	+
BB0465	+	+	+	-	+	-	-	+	+	+
BB0543	-	+	+	+	+	-	-	+	+	+
BB0564	-	+	+	+	+	-	-	-	-	+
BB0624	+	+	-	+	+	-	-	-	-	+
BB0662	+	+	-	+	+	-	-	+	-	+
BB0743	+	+	-	+	-	+	-	+	-	+
BB0761	+	+	-	+	-	+	-	-	-	+
Group 4 (3/6)										
BB0032	+	+	-	+	-	-	-	-	+	+
BB0039	+	-	-	+	-	+	-	+	+	+
BB0043	+	-	-	+	+	-	-	-	+	+
BB0058	+	-	-	+	-	+	+	+	+	+
BB0102	+	+	-	+	-	-	+	+	-	-
BB0125	+	+	-	+	-	-	-	-	+	-
BB0165	+	+	-	+	-	-	-	-	-	+
BB0322	+	+	+	-	+	-	-	-	-	+
BB0352	+	+	-	+	-	-	+	+	-	+
BB0405	-	+	+	-	+	-	-	+	+	+
BB0406	+	+	+	-	-	-	+	+	-	+
BB0458	+	+	-	+	-	-	-	+	-	+
BB0546	+	+	-	+	-	-	-	-	+	+
BB0735	+	+	-	+	-	-	-	+	+	+
BB0790	+	-	-	+	-	+	-	-	+	+

^*a*^Proteins are grouped according to the number of OM prediction programs that predict localization to the OM or β-barrel formation. The outer membrane protein prediction programs included CELLO, PSORTb, HHOMP, TMBETADISC-ACC, PRED-TMBB and BOMP

^*b*^Signal sequences were predicted by the programs SignalP 3.0, PrediSi, and Signal-CF. Signal sequences were also manually inspected by hydrophilicity plots generated according to the methods of Kyte and Doolittle

Importantly, we identified several known borrelial OMPs by the computational framework utilized suggesting that the algorithm was effective at identifying OMPs encoded in the *B. burgdorferi* B31 genome. Within the first group of candidate OMPs (i.e., Group 1; sequences predicted to be OMPs by all six programs utilized in the study) were two borrelial proteins previously identified and characterized as OMPs: BamA [39] and the pore-forming OMP DipA [41] (Table 1). Furthermore, we also identified in Group 2 (predicted as an OMP by 5 of 6 programs) the borrelial OMP P66 (BB0603), a known adhesin that binds β_3_-integrins [34–37], has porin activity [50], and adopts a β-barrel conformation [51]. In Group 3 (predicted as an OMP by 4 of 6 programs), the known OMP BesC [33] was also identified using the algorithm outlined. Notably, Pal and colleagues previously identified borrelial proteins associated with OM complexes from *B. burgdorferi* B31 [53]. When we compared our list of candidate OMPs with the list of proteins detected in the borrelial OM in this prior report, we found numerous similarities, including the known borrelial OMPs DipA, BamA, P66, and BesC as well as the hypothetical proteins BB0543, BB0662, BB0125, BB0405, and BB0406.

In addition to the known borrelial OMPs identified in Group 1, two uncharacterized hypothetical proteins, BB0794 and BB0838, were also predicted as potential candidate OMPs in this first group (Table 1). The protein BB0794 is encoded by an ORF situated directly upstream of BamA, which is a known OMP. BB0794 has a conserved DUF490 domain which has been reportedly found in TamB orthologs [68]. While TamB is actually an inner membrane protein that is part of the translocation and assembly module (TAM), the C-terminus of TamB is predicted to have β-sheet topology and has been predicted by numerous computer-based prediction programs to be similar to a β-barrel OMP [69–72]. Given this previous work with TamB from other organisms, it is not surprising that BB0794 was identified as a candidate OMP in our studies. BB0838, also identified in Group 1, is encoded by an ORF downstream of *B. burgdorferi* B31 *uvrA* and *uvrB*, which encode proteins involved in nucleotide excision repair [73, 74]. Interestingly, BB0838 has a conserved LptD (lipopolysaccharide transport protein D) domain, which is the known OM, β-barrel component of the translocation complex that transports lipopolysaccharide (LPS) to the cell surface [75, 76].

Unlike the first three groups, no fully characterized borrelial OMPs were included in the Group 4 list of candidates that were predicted to be an OMP by 3 of the 6 localization and topology programs. It should be noted, however, that BB0405 was identified in Group 4, and we have previously reported that BB0405 is likely localized to the OM, is amphiphilic, and appears to be surface-exposed [22, 39]. The paralogous BB0406 (59 % sequence similarity to BB0405), encoded by the ORF immediately downstream of BB0405, also was included in this group of candidates.

### *B. burgdorferi* B31 BB0838 is a surface-exposed, amphiphilic protein

**Fig. 2:**
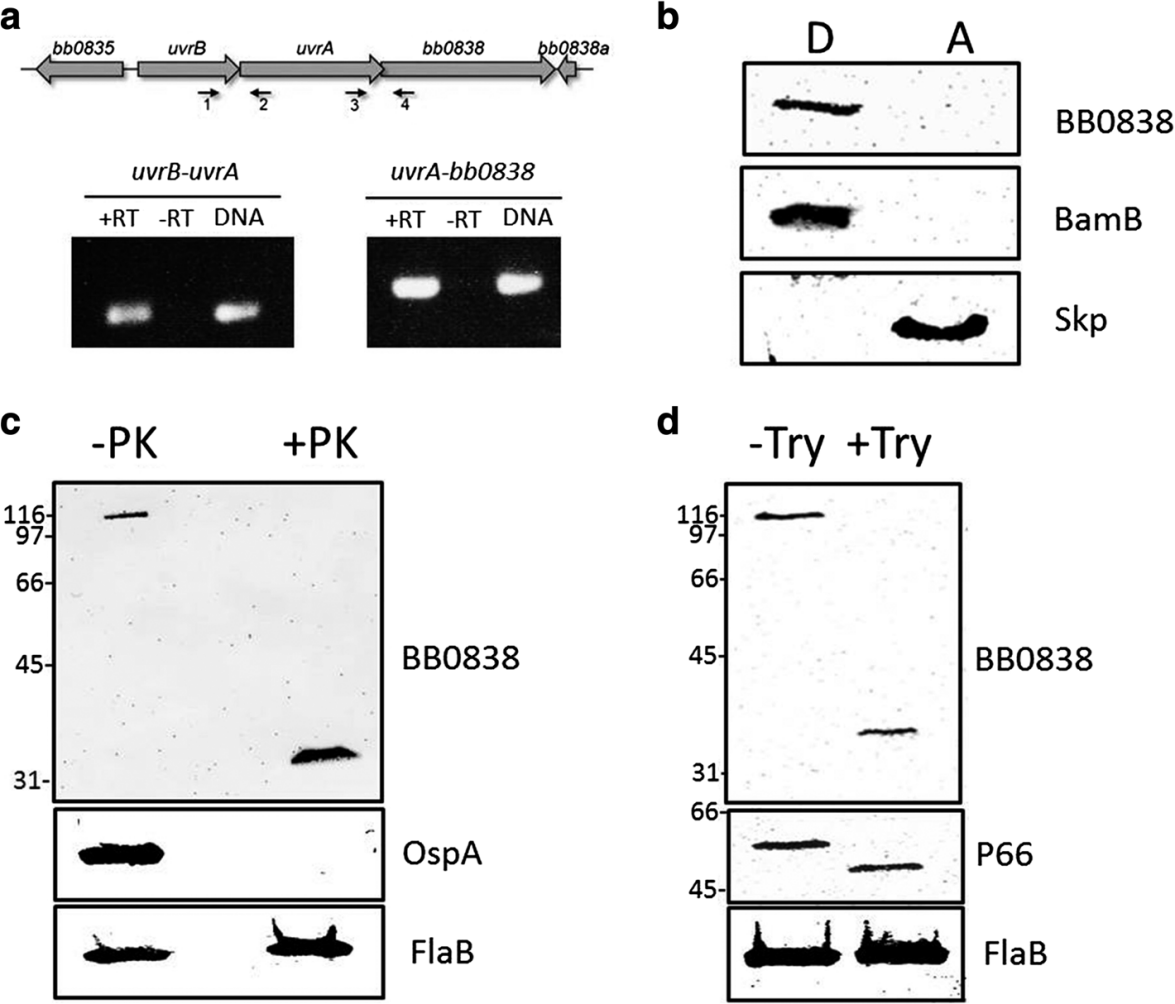
*B. burgdorferi* B31 BB0838 is surface localized and amphiphilic. **a**. *bb0838* is in an operon with *uvrA* and *uvrB*. Schematic of the *uvrB*, *uvrA*, and *bb0838* operon is shown in the top panel. Total RNA was isolated from *B. burgdorferi* B31 cells and used for RT-PCR using primer pairs listed in Table 3. Primer pairs were used that amplify a region traversing *uvrB* and *uvrA* (primers 1 and 2, left panel) and *uvrA* and *bb0838* (primers 3 and 4, right panel). A negative control lacking RT was used as template for the RT-PCR (−RT) as was as a positive control in which genomic DNA instead of cDNA was used as template (DNA). **b**. Triton X-114 phase partitioning of *B. burgdorferi* B31 whole-cell lysates was performed to separate soluble, aqueous (A) phase proteins from amphiphilic, detergent (D) phase proteins. Aqueous and detergent fractions were separated by SDS-PAGE and immunoblotted with anti-BB0838 peptide antibodies. Equivalent fractions were also immunoblotted with anti-BamB and anti-Skp antibodies as detergent-enriched and aqueous-enriched controls, respectively. **c-d**. Whole-cell lysates were washed and incubated with (**c**) proteinase K (PK) or (**d**) trypsin. Samples were then immunoblotted with BB0838 peptide antibodies to assess surface degradation of BB0838. Equivalent samples were also immunoblotted with OspA antibodies or P66 antibodies for PK and trypsin experiments, respectively, to control for protease activity and with antibodies that recognize the periplasmic protein FlaB to ensure that the OM remained intact throughout the proteolysis experiments. Molecular weight standards (in kDa) are shown at left

To confirm that the computational method utilized to screen for candidate proteins did, in fact, correctly identify novel OMPs from *B. burgdorferi,* we next examined the cellular localization of specific candidate OMPs identified. As mentioned above, Group 1 contained the candidate BB0838 (Table 1) that encodes an LptD domain, which is a characteristic of LptD proteins known to contain a C-terminal β-barrel region. Consistent with BB0838 being a putative OMP, when BB0838 was modeled without a specified template using the I-TASSER [77–79] and SPARKS-X [80] programs, LptD proteins were identified as the top hit for structural modeling similarities in all cases. BB0838 is predicted to be approximately 120 kDa and is encoded downstream of *uvrB* and *uvrA*. Given that *uvrB*, *uvrA*, and *bb0838* were all located in close proximity on the chromosome, with the *uvrA* and *bb0838* ORFs overlapping, we first examined whether these genes were part of an operon using RT-PCR. This analysis confirmed that *uvrB*, *uvrA*, and *bb0838* are encoded on the same transcript (Fig. 2a). We next examined whether BB0838 is membrane-associated by performing Triton X-114 phase partitioning studies to separate membrane proteins from soluble cytoplasmic and periplasmic proteins [81]. BB0838 partitioned into the detergent-enriched fractions after Triton X-114 phase partitioning of *B. burgdorferi* B31 whole-cell lysates suggesting that BB0838 has the properties expected of an amphiphilic membrane protein (Fig. 2b). For phase partitioning experiments, the lipoprotein BamB [42] served as a membrane protein control, while the soluble Skp protein partitioned into the aqueous phase as expected (Fig. 2b).

To assess whether BB0838 is surface-exposed, *B. burgdorferi* B31 cells were treated with protease proteinase K (PK). Immunoblot analysis of PK treated cells revealed that BB0838 is at least partially susceptible to PK degradation suggesting that the protein has surface-exposed regions (Fig. 2c). In fact, PK treatment resulted in a breakdown of the full length protein of approximately 120 kDa to a smaller protein band of approximately 33 kDa (Fig. 2c). To further characterize the potential surface-exposed regions of BB0838, we also treated cells with the protease trypsin, which specifically targets the carboxyl side of lysine and arginine residues for proteolysis. Trypsin proteolysis resulted in a degradation pattern similar to PK treatment; however, the breakdown product was slightly larger than the protein band detected when cells were treated with PK (Fig. 2d). For both PK and trypsin assays, known PK and trypsin sensitive proteins OspA and P66, respectively, were degraded in the presence of the enzyme as expected (Fig. 2c-d). Furthermore, the periplasmic FlaB protein was not degraded in either experiment and served as a control for membrane integrity throughout the proteolysis studies (Fig. 2c-d).

### *B. burgdorferi* B31 BB0405 and BB0406 are amphiphilic and OM-localized

**Fig. 3:**
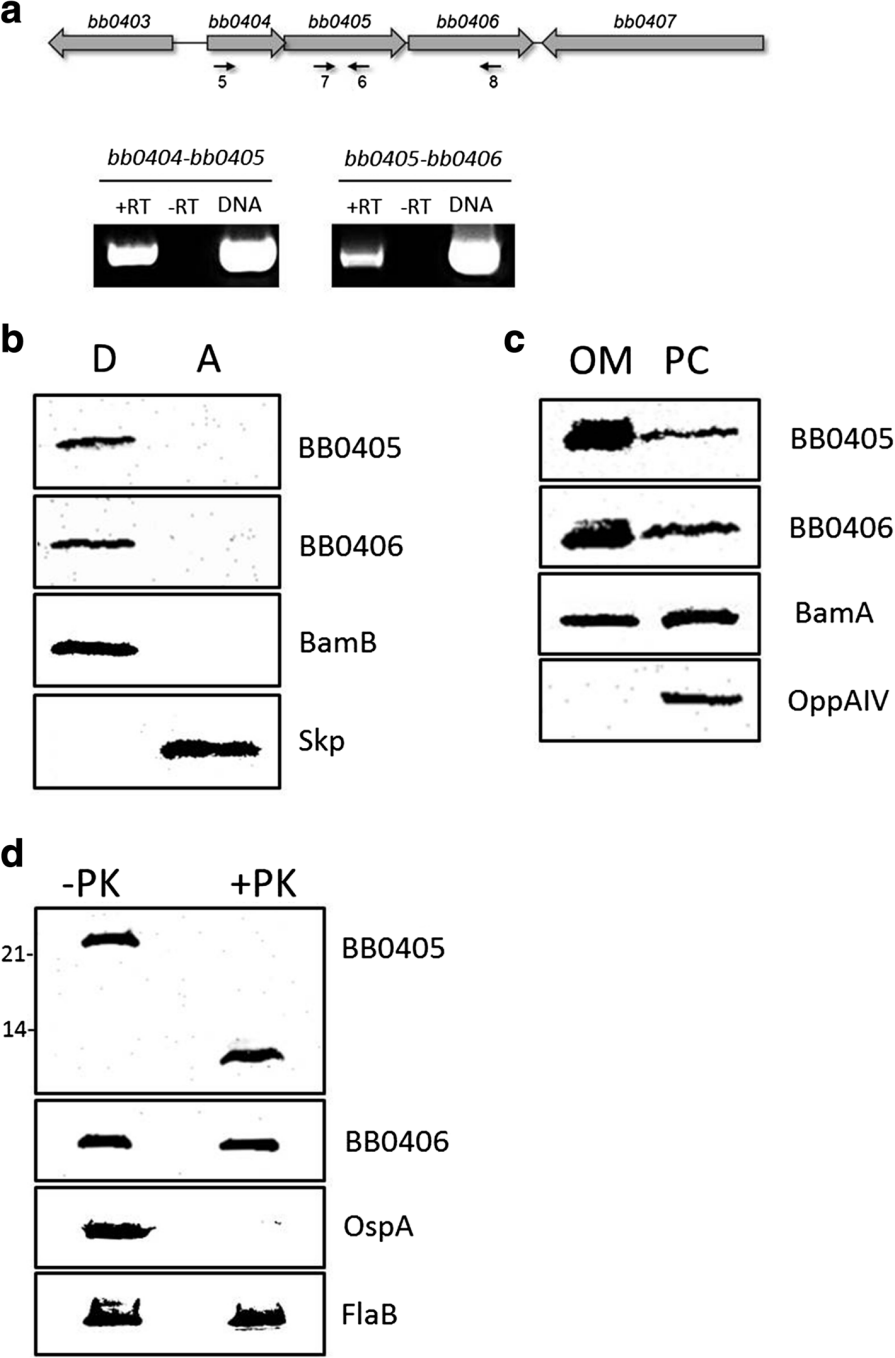
*B. burgdorferi* B31 BB0406 is amphiphilic and OM-associated. **a**. *bb0404*, *bb0405*, and *bb0406* are transcribed as an operon. Schematic of the *bb0404*, *bb0405*, and *bb0406* operon is shown in the top panel. Total RNA was isolated from *B. burgdorferi* B31 cells and used for RT-PCR using primer pairs listed in Table 3. Primer pairs were used that amplify a region traversing *bb0404* and *bb0405* (primers 5 and 6, left panel) and *bb0405* and *bb0406* (primers 7 and 8, right panel). A negative control without RT was used as template for the RT-PCR (−RT) as was as a positive control in which genomic DNA instead of cDNA was used as template (DNA). **b**. Triton X-114 phase partitioning of *B. burgdorferi* B31 whole-cell lysates were performed to separate aqueous-enriched (A) proteins from detergent-enriched (D) proteins. Aqueous and detergent fractions were separated by SDS-PAGE and immunoblotted with rat anti-BB0405 and rat anti-BB0406 antibodies. As controls, equivalent fractions were also immunoblotted with antibodies directed against the detergent-soluble lipoprotein BamB and the soluble, periplasmic protein Skp. **c**. Outer membrane (OM) and protoplasmic cylinder (PC) fractions were isolated from *B. burgdorferi* B31. Subsequently, OM and PC fractions were immunoblotted with rat anti-BB0405 and anti-BB0406 antibodies. Equivalent membranes were also subjected to immunoblot with BamA and OppAIV antibodies. **d**. Whole-cell lysates were washed and incubated with proteinase K (PK). Samples were then immunoblotted with BB0405 or BB0406 antibodies to assess surface degradation of the protein. Equivalent samples were also immunoblotted with OspA antibodies to control for protease activity and with antibodies that recognize the periplasmic protein FlaB to ensure that the OM remained intact throughout the proteolysis experiments

Both *B. burgdorferi* BB0405 and BB0406 were detected in Group 4 of the candidate OMP list (Table 1). The prior observations suggesting that BB0405 is surface- and OM-localized [22, 39], combined with the observation that BB0405 and BB0406 are detected in OM vesicles isolated from *B. burgdorferi* B31 [53], strongly suggested that Group 4 also contained novel OMPs that warranted further characterization. Thus, we next examined the cellular localization and physicochemical properties of the two Group 4 proteins BB0405 and BB0406. We first looked more closely at the genomic organization of *bb0405* and *bb0406* and found that *bb0405* overlaps with the upstream gene *bb0404*. Therefore, we examined whether these genes were co-transcribed with the *bb0404*. RT-PCR utilizing primers that amplified regions traversing *bb0404* and *bb0405* as well as *bb0405* and *bb0406* revealed that all three genes are co-expressed in a single transcript (Fig. 3a). Triton X-114 phase partitioning experiments revealed that both BB0405 and BB0406 are membrane proteins as they both partitioned into the detergent-enriched phase (Fig. 3b). Equivalent fractions were also immunoblotted with anti-BamB antibodies and anti-Skp antibodies which served as membrane and soluble protein controls, respectively (Fig. 3b). Since phase partitioning experiments suggested BB0405 and BB0406 are amphiphilic, we next examined whether they are localized to the borrelial OM. OMs and protoplasmic cylinders (PC) were enriched from *B. burgdorferi* B31 cells and subsequently immunoblotted (Fig. 3c). BB0405 and BB0406 were detected in the OM fractions indicating both are OMPs (Fig. 3c). BamA, a known *B. burgdorferi* B31 OMP, was detected in the OM as expected, while the inner membrane lipoprotein OppAIV was detected only in the PC fraction indicating the OM fraction was highly enriched (Fig. 3c).

Given that BB0405 and BB0406 are OM-localized and display the properties expected of OMPs, we next examined whether BB0405 and BB0406 are also surface-exposed. PK surface-localization assays were performed and PK treated and sham treated cell lysates were immunoblotted with BB0405 and BB0406 antibodies. Surprisingly, while BB0405 was partially PK sensitive with only an ~10 kDa region of 405 being protected from the PK degradation, BB0406 was entirely PK resistant (Fig. 3d). This suggested that BB0406 is either not surface-exposed or is protease resistant. OspA served as a positive control in the PK experiments (Fig. 3d). To confirm that the OM remained intact during PK surface proteolysis, equivalent membranes were also immunoblotted with anti-FlaB antibodies to demonstrate that the periplasmic FlaB protein was not exposed to PK degradation (Fig 3d).

### *B. burgdorferi* B31 BB0405 and BB0406 associate with and form pores in LUVs

**Fig. 4:**
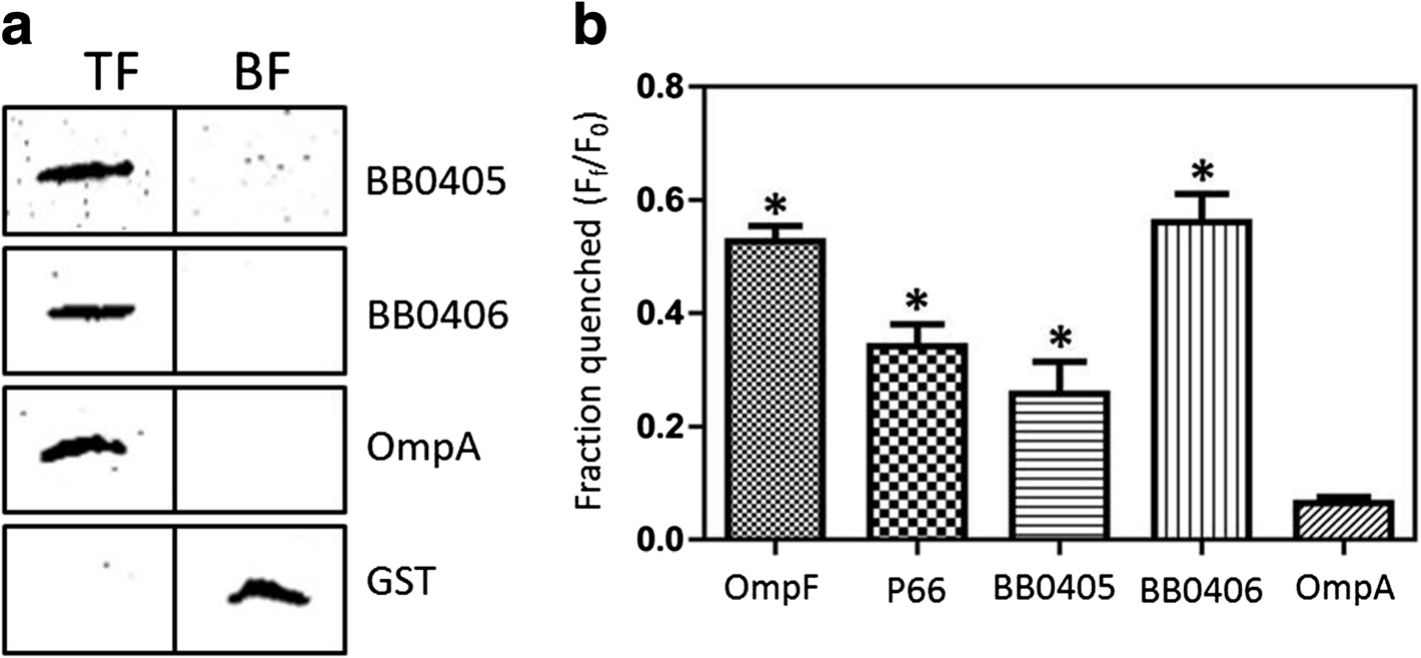
**a**. Liposomes simulating the *B. burgdorferi* B31 OM were incubated with recombinant BB0405, BB0406, *E. coli* OmpA, or GST and then separated on discontinuous sucrose gradients. Gradient fractions were collected from the top (TF) and bottom (BF) and subjected to immunoblot with appropriate antibodies. **b**. Liposomes loaded with the fluorophore Tb(DPA)_3_^3−^ were incubated with recombinant *B. burgdorferi* B31 BB0405, BB0406, P66, *E. coli* OmpF, or *E. coli* OmpA in buffer supplemented with EDTA, and fluorophore efflux was measured as quenched fluorescence. Three independent experiments were compared and each bar represents the mean ± S.E. (error bars). Statistical significance compared with *E. coli* OmpA is indicated with * (*p* < 0.05)

Since both BB0405 and BB0406 are associated with the OM, we next wanted to determine if these proteins could integrate into lipid bilayers and form pores. To examine whether folded recombinant *B. burgdorferi* BB0405 and BB0406 could incorporate into large unilamellar vesicles (LUVs) that were generated to mimic the phospholipid content of the *B. burgdorferi* B31 OM [82], LUVs were incubated separately with folded recombinant BB0405 or BB0406. The mixture was then separated on discontinuous sucrose gradients to separate the liposome-containing top fraction (TF) from the bottom fraction (BF), which contains unincorporated protein. Fractions were then subjected to immunoblot analysis with antisera directed against BB0405 or BB0406. Both proteins were detected in the TF suggesting that BB0405 and BB0406 were able to incorporate into liposomes (Fig. 4a). When experiments were also performed with folded recombinant *E. coli* OmpA, a well characterized OM protein [83], OmpA was also detected in the TF (Fig. 4a). As expected, the soluble GST protein was unable to incorporate into LUVs and was thus only detected in the BF (Fig. 4a).

We next assessed whether BB0405 and BB0406 had porin like properties and could form pores in LUVs using a pore formation assay to measure efflux of fluorophore Tb(DPA)_3_^3−^ from liposomes incubated with the folded recombinant protein. When liposomes were loaded with folded BB0405 or BB0406 protein, fluorophore efflux from the loaded liposomes was detected (Fig. 4b), indicating that both proteins were capable of forming pores. Escape of the fluorophore was also detected when liposomes were incubated with the known pore-forming *E. coli* protein OmpF as well as the *B. burgdorferi* B31 P66 protein (Fig. 4b), which we have previously shown forms pores in LUVs [51]. In contrast, *E. coli* OmpA, which occurs mostly in a closed conformation, did not efficiently generate pores in the LUVs, and thus only low levels of efflux was detected (Fig. 4b), which is entirely consistent with previous reports [84, 85]. Taken together these studies suggest that both BB0405 and BB0406 are OM-localized proteins capable of forming pores.

### OMP candidate proteins from Lyme disease and relapsing fever *Borrelia*

After demonstrating that the computational analysis could accurately predict novel *B. burgdorferi* OMPs, we expanded our computational analysis to predict OMPs from spirochetes belonging to both the Lyme disease- and relapsing fever-causing groups (see Table 2). In broadening the scope of the study, we looked specifically for OMPs conserved among all *Borrelia* genospecies that could be potential universal targets for future vaccine studies. Furthermore, novel OMPs conserved among the *Borrelia spp*. could be important virulence determinants and relevant in spirochete physiology. For this analysis, all chromosomally encoded proteins from twelve Lyme disease *Borrelia* including genospecies *B. burgdorferi*, *B. garinii*, *B. afzelii*, *B. valaisiana*, and *B. bissettii* and seven relapsing fever *Borrelia* including *B. duttonii*, *B. crocidurae*, *B. parkeri*, *B. miyamotoi*, *B. turicatae*, *B. recurrentis*, and *B. hermsii* were subjected to the same computational analysis as outlined in Fig. 1. All genomes analyzed as well as accession numbers are listed in Table 2, and the data collected for all ORFs are presented in Additional file 1: Table S1 and summarized in Table 2. After collecting the data for all chromosomally encoded protein sequences from nineteen *Borrelia* organisms, candidate OMPs from each genome were identified using the same process that was described above to identify candidate OMPs from *B. burgdorferi* B31. The candidate OMPs identified from the chromosomes of all nineteen species are listed in Additional file 2: Table S2.

**Table 2:** *Borrelia* genomes analyzed

Genome analyzed	Accession number	Total number of proteins	Predicted lipoproteins	Predicted proteins with trans-membrane domains	Predicted OM/β-barrel proteins
Lyme disease spirochetes					
*Borrelia burgdorferi* B31	AE000783.1	797	36	152	41
*Borrelia burgdorferi* ZS7	CP001205.1	808	34	151	41
*Borrelia burgdorferi* N40	CP002228.1	809	34	155	39
*Borrelia burgdorferi* JD1	CP002312.1	823	39	154	41
*Borrelia burgdorferi* CA382	CP005925.1	819	34	153	39
*Borrelia garinii* BgVir	CP003151.1	826	33	157	40
*Borrelia garinii NMJW1*	CP003866.1	813	28	150	35
*Borrelia garinii Pbi*	CP000013.1	825	33	158	38
*Borrelia afzelii HLJ01*	CP003882.1	892	27	161	42
*Borrelia afzelii PKo*	CP002933.1	824	31	159	36
*Borrelia valaisiana VS116*	ABCY02000001.1	832	31	163	37
*Borrelia bissettii DN127*	CP002746.1	816	28	157	42
Relapsing fever spirochetes					
*Borrelia duttonii Ly*	CP000976.1	820	36	164	36
*Borrelia crocidurae str. Achema*	CP003426.1	864	30	162	37
*Borrelia parkeri HR1*	CP007022.1	825	36	158	27
*Borrelia miyamotoi LB-2001*	CP006647.2	808	27	156	27
*Borrelia turicatae 91E135*	CP000049.1	818	37	164	32
*Borrelia recurrentis A1*	CP000993.1	800	32	157	34
*Borrelia hermsii HS1*	CP000048.1	819	35	159	40

**Fig. 5:**
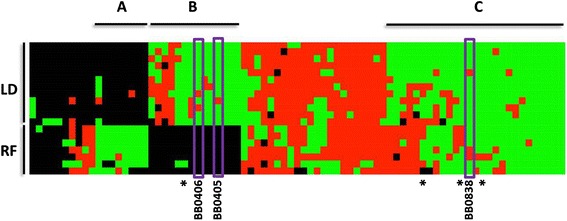
To assess the value of these predictions to identify putative vaccine immunogens, predicted OMPs from each of the *Borrelia* genomes were organized into orthologous clusters, and the resulting information was presented as a heat map. In this analysis, clusters of *Borrelia* proteins predicted to be OMPs are represented as green blocks, while clusters of *Borrelia* proteins orthologous to these, but not passing all OMP prediction filters are represented in red. If a cluster of orthologous proteins was not encoded in a given borrelial genome, this was represented as a black block. These data were then subject to a two-way hierarchical clustering, by genome and by orthologous protein cluster. From this analysis, three groups of predicted OMPs were identified: Group **a** represents predicted borrelial OMPs unique to relapsing fever (RF) organisms, Group **b** represents predicted borrelial OMPs unique to Lyme disease (LD) pathogens and Group **c** includes predicted OMPs shared by both LD and RF pathogens. The candidate OMPs analyzed in this study are indicated with purple boxes corresponding to BB0405, BB0406, and BB0838. Asterisks (*) indicate known borrelial OMPs identified in the study including BamA, P66, BesC, and DipA. A larger version of this figure with cluster IDs and genome designations is provided in Additional file 4: Figure S1

The global analysis of numerous *Borrelia* genomes allowed us to determine which candidate OMPs are conserved among the various species analyzed. To do this, using CD-hit, we first sorted all protein sequences into orthologous clusters [86] [Additional file 3: Table S3]; these results were then used to construct a heat map [87] that encompassed candidate OMPs specific to relapsing fever spirochetes, Lyme disease spirochetes, or both Lyme disease and relapsing fever spirochetes (Fig. 5 and Additional file 4: Figure S1; predicted OMPs in green). The cluster analysis also included protein-coding sequences in these genomes that were not predicted to be OMPs, but were nevertheless orthologous to one or more predicted OMPs (Fig. 5; orthologs in a cluster not predicted to be OMPs are indicated in red). The heat map also indicates if no ortholog was detected in the cluster for a given genome (Fig. 5; indicated in black). In some clusters, almost all of the orthologs within the cluster were predicted to be OMPs with only a few orthologs not passing the computational analysis used for OMP prediction. Apparent false negative predictions could be identified as the rare ortholog(s) within a single cluster that was not predicted to be an OMP while all of the other cluster members were predicted to be OMPs. Conversely, apparent false positive predictions also were observed that could be visualized as the rare ortholog(s) predicted to be an OMP when the majority of the members of the unique cluster were not predicted OMPs. For instance, BB0838 was predicted to be an OMP by sixteen of the nineteen genomes analyzed from both Lyme disease and relapsing fever species, suggesting that it is likely an OMP in all genomes and there were three genomes with false negative predictions (Fig. 5). The BB0405 and BB0406 orthologs were predicted to constitute clusters of candidate OMPs specific for Lyme disease causing spirochetes. One BB0405 and two BB0406 orthologs were predicted not to be OMPs, suggesting these are false negative predictions since 11/12 and 10/12 Lyme disease associated genomes predicted BB0405 and BB0406 to be an OMP, respectively (Fig. 5). While relapsing fever spirochetes do encode BB0405 and BB0406 orthologs, those protein sequences did not cluster with the orthologs from the Lyme disease spirochetes according to the parameters used in this study. Collectively, these data provide a group of candidate OMPs that are conserved among various species of *Borrelia* spirochetes and can be examined as potential OMPs and virulence factors in future studies.

## Discussion

Given that Lyme disease and relapsing fever spirochetes are extracellular pathogens, the surface of these spirochetes and the proteins localized to their OM provide the interface between these pathogens and their various hosts during infection. It also has been established that humoral immunity provides protection against these infections; therefore, borrelial surface proteins have been the target of much study with regards to identifying new vaccine targets. To date, most vaccine studies have focused on the immunogenic surface lipoproteins [12, 25, 88–93] with much less emphasis being placed on integral OMPs localized to the surface of the organism. This is due in part to the fact that surface lipoproteins are highly immunogenic and highly abundant, which has made lipoproteins much easier to identify and characterize than the borrelial OMPs. In this study, we sought to identify novel OMPs from *Borrelia*. Given that computer-based algorithms do not always accurately predict protein structure, we employed a consensus strategy in which we relied on six different algorithms that predict both OM localization and β-barrel conformation to identify candidate borrelial OMPs [58–63]. By using this stratified consensus approach, we were able to prioritize candidate OMPs based on the number of programs predicting that a specific sequence encoded a novel OMP. We curated our list by eliminating lipoproteins using a lipoprotein prediction algorithm specific for spirochetes [55] and proteins containing transmembrane domains [56, 57] that are indicative of inner membrane proteins. Moreover, we only accepted sequences with an N-terminal signal peptide as would be required for translocation across the bacterial inner membrane, and we again instituted a consensus approach using four different programs to predict signal peptides [64–67]. Importantly, we identified several known borrelial OMPs by the computational framework utilized including BamA, BesC, P66, and DipA, and, of the well-characterized OMPs identified thus far, only the known borrelial OMP P13 was not identified in our study. In a previous study, chromosomally encoded proteins from all sequenced bacterial genomes including the *Borrelia* spirochetes were analyzed by the TMBB algorithm which aims to identify β-barrel, OMPs [94]. Using this single method, 26 candidate *B. burgdorferi* B31 OMPs were identified of which eight were orthologous to non-OMPs. The study presented herein, however, identified 41 candidate OMPs further supporting the notion that the consensus approach utilized in these studies is an improvement from relying on a single algorithm.

Notably, we were able to further prioritize our list of candidate OMPs by examining and comparing the output generated from the chromosomes of nineteen different Lyme disease and relapsing fever organisms. With this approach, candidate OMPs could be further verified by the overall likelihood that the sequence was predicted to be an OMP in numerous other related borrelial organisms. In fact, potential false-positive or false-negative predictions in a single spirochetal species or strain can easily be identified by comparing the OMP predictions for proteins in the same cluster from many different organisms as outlined here. Furthermore, this more global approach will allow future studies to focus on sequences predicted to be OMPs in numerous species that are considered viable vaccine candidates based on their overall sequence homology and tendency to cluster together according to the algorithm utilized in this study. The cluster analysis demonstrated that there is a large group of predicted OMPs that are shared among the genomes of all currently sequenced *Borrelia* spp. analyzed (Fig. 5, Group C), which potentially represent vaccine candidates that could protect against both Lyme disease and relapsing-fever infections. This is an important addition that could not only enhance vaccine development for various spirochete diseases, but this system could also be used for other groups of human pathogens given that one of the major caveats in vaccine development has been generating protective immune responses against multiple strains or species.

While it is likely that some of the candidates identified using the computational algorithm outlined herein are not OMPs, the physicochemical data provided for BB0405, BB0406, and BB0838 provide a robust proof of principle that the algorithm can accurately identify novel OMPs. Interestingly, a larger domain of the C-terminal region of BB0838 was protected from degradation when organisms were treated with trypsin as compared to proteinase K. This type of proteolysis data would be entirely consistent with a large extracellular loop that contains a trypsin susceptible lysine or arginine residue but is otherwise fully susceptible to proteinase K degradation. Consistent with this notion, the *B. burgdorferi* OMP P66 also has been shown in previous studies to be partially sensitive to both proteinase K and trypsin with a lysine residue in a surface loop that is uniquely susceptible to trypsin [51]. Interestingly, *bb0838* was found to be co-transcribed with *uvrA* and *uvrB*. UvrA and UvrB along with UvrC and UvrD are members of the nucleotide excision repair (NER) pathway which was previously shown to be the only borrelial DNA repair pathway that functions to repair DNA in response to UV light damage [73, 74]. In fact, *B. burgdorferi* spirochetes appear to have a reduced number of DNA repair enzymes [11, 20]. Why a potential borrelial OMP such as BB0838 would be co-expressed with two components of the NER pathway is unclear. It is worth noting, however, that this gene arrangement and genomic organization was conserved among all Lyme disease- and relapsing fever-causing spirochetes examined. This conservation in gene organization and, by correlation, co-expression pattern of *uvrAB* and *bb0838* and its various orthologs suggests this happened prior to the divergence of the various *Borrelia* spp. Whether BB0838 can incorporate into liposomes and/or forms a pore remains to be determined. Such experiments with BB0838 could not be performed due to the large size of the full-length native protein and our inability to generate a full-length recombinant protein that was not lethal when expressed in *E. coli.*

BB0406 also was observed to be a novel OMP that had not previously been identified from *B. burgdorferi* or any other *Borrelia* spp. to date. As previously suggested, BB0405 was confirmed to be a surface-exposed OMP [22, 39]. While we demonstrated that BB0405 and BB0406 are OM-localized and capable of forming pores, BB0406 was found to be resistant to proteinase K degradation. Complete and partial resistance to protease has been reported for many other bacterial OMPs [30, 39, 95–98], so it was not entirely surprising that a borrelial OMP also is protease resistant. Given that we have now shown that BB0406 is a potential porin, it seems most likely BB0406 has either very little surface exposure or the protein is possibly protected from degradation by interacting with other surface OMPs or the abundant surface lipoproteins as was previously shown for the known OMP P66 [99]. *Borreliae* are known to have a relatively small genome as compared to other bacterial organisms and are thus dependent on the uptake of nutrients from the host environment. This highlights the importance of proteins such as porins for the survival of the spirochete throughout the organism’s life cycle. Yet, a limited number of porins have been identified in *B. burgdorferi* [41, 50, 100] despite the observation that in black lipid bilayer experiments using borrelial OM fractions there are numerous pore-forming proteins that have not been identified [101]. While BB0405 and BB0406 do form pores, we have not shown that these proteins form β-barrels as was predicted by the computer algorithms utilized in this study. Crystal structures will need to be resolved in order to definitively determine whether these proteins are β-barrels as predicted by the algorithm. However, recent reports have examined the structural conformation of both the *B. afzelii* BB0405 ortholog (BaPKo_0422) [102, 103] and the *B. garinii* BB0406 ortholog (BG_0408) using small-angle X-ray scattering [103]. Notably, in these reports, it was demonstrated that both proteins form structures entirely consistent with an 8-stranded β-barrel further supporting the notion that BB0405 and BB0406 fold into β-barrels in the borrelial OM.

Among previously characterized pore-forming OMPs in *B burgdorferi*, P13 and P66 are both known to be immunogenic and P66 has been shown to be at least partially protective in mice challenged with *B. burgdorferi* B31 [104–106]. Prior studies have shown that OMPs P66 and BesC are also required for establishing infection in mice [33, 107]. Along these lines, we also previously observed that antibodies recognizing the putative OMP BB0405 were bactericidal and that nonhuman primates infected with *B. burgdorferi* elicit a specific antibody response against BB0405 [22]. In other studies, we also have determined that a mutant *B. burgdorferi* strain lacking BB0405 and BB0406 is unable to establish an infection in mice, suggesting that one or both of these proteins is also essential for mammalian infection (Shrestha, Kenedy, and Akins, unpublished observations). Whether the inability of the BB0405/406 mutant to infect mice is dependent on the porin function of these proteins is unknown at this time. Notably, BG0407 the *B. garinii* BB0405 ortholog and BAPK0422 the *B. afzelii* BB0405 orthlolg have both been shown to bind human Factor H [102, 108]. Binding of Factor H by bacteria inhibits the alternative pathway of complement and is a method of immune evasion for pathogenic organisms. Whether *B. burgdorferi* BB0405 also binds Factor H is unknown at this time. BB0838 also may play an important role in the infectious life cycle of *B. burgdorferi*. When global transposon mutagenesis of *B. burgdorferi* was performed by Norris and colleagues, no transposon insertions were identified within the *bb0838* gene [109, 110], suggesting it is an essential protein.

## Conclusions

The computational and bioinformatics studies presented have identified novel OMPs from *B. burgdorferi* (BB0838 and BB0406) and confirmed that BB0405 also is a *B. burgdorferi* OMP. Furthermore, BB0405 and BB0406 were shown to have pore forming properties, suggesting they may play a role in allowing *Borrelia* spp. to sample and respond to environmental changes. In addition to a better catalog of candidate OMPs from various borrelial species, the computational framework utilized here could also help to identify new vaccine candidates for future studies. As it relates to vaccine-development, the overall clustering observed in Fig. 5 is provocative and points out specific candidate OMPs that could be targeted specifically for future Lyme disease or relapsing fever vaccine studies. Most important, however, is the fact that it may now be possible to identify potential vaccine candidates that could target both of these important human diseases with a single or multi-subunit vaccine consisting of OMPs shared by both groups. Apart from this applied aspect of vaccine development, basic mechanisms of molecular pathogenesis for this wide array of spirochetes could also be revealed in the various OMP protein clusters, which could imply functional specialization of specific OMPs that have evolved to support specific activities by Lyme-disease or relapsing-fever spirochetes. Further studies will be required to delineate among the proteins identified as to which are actual OMPs and which may be false positives. Finally, while the focus of our study was limited to *Borrelia* spp., it seems self-evident that a similar strategy could be used to identify OMPs from bacteria other than spirochetes to identify new vaccine targets for many different human diseases.

## Methods

### Computational framework for identifying candidate *Borrelia* outer membrane proteins (OMP)

Candidate OMPs were predicted according to the methods outlined in Fig. 1 and server URLs are listed in Additional file 5: Table S4. A summary of the servers utilized in this study was outlined previously [54]. The *Borrelia* genomes examined in the study are listed in Table 2 along with accession numbers. First, protein sequences from each open reading frame on each *Borrelia* chromosome was analyzed to determine if the protein was predicted to be a lipoprotein using the SpLip algorithm which was developed to specifically identify the unique characteristics of spirochaetal lipoproteins and was kindly provided by the authors [55]. Next, sequences were analyzed to determine if the protein was predicted to contain transmembrane α-helices by the Phobius server [56] and the TMHMM server [57]. Outer membrane (OM) localization was next predicted by CELLO [58] and PSORTb 3.0 [59]. The following servers were utilized to predict β-barrel conformation: HHOMP [60], TMBETADISC-AAC [61], PRED-TMBB [62, 111], and BOMP [63]. After all sequences were examined by the above algorithms, a candidate OMP list was generated according to the following steps: (1) any protein predicted to be a lipoprotein was discarded, (2) any protein predicted to contain transmembrane α-helices by either Phobius or TMHMM servers were discarded, (3) proteins were retained if they were predicted to be OM-localized by either CELLO or pSORTb 3.0, and (4) proteins were retained if they were predicted to be a β-barrel by at least one of the four or two of the four β-barrel prediction programs depending on if the sequence was predicted to be OM-localized by two or one of the OM-localization programs, respectively. The remaining proteins were manually analyzed to remove any proteins orthologous to proteins that are not OMPs (i.e., proteins annotated to be orthologous to characterized cytoplasmic, inner membrane, or periplasmic proteins or any lipoproteins). Furthermore, the remaining sequences were subjected to analysis for an N-terminal signal peptide by SignalP 3.0 [64], PrediSi [65]), Signal-CF [66], and manually inspected for signal sequences using hydrophilicity plots according to the methods of Kyte and Doolittle [67]. Sequences were only retained if the protein was predicted by at least one signal peptide prediction program to have a N-terminal signal peptide. Finally, candidate OMPs were grouped by the number of programs predicting OM localization and β-barrel conformation. Proteins were removed from the candidate OMP list if the sequence was not predicted to be OM localized and have β-barrel conformation by three of the following six algorithims: CELLO, pSORTb, HHOMP, TMBETADISC-AAC, PRED-TMBB, and BOMP.

To identify clusters of orthologous sequences for predicted OMPs in each genome, the protein sequences were clustered using CD-hit [86] with cut-off parameters for percent identity and percent length equal to .50 and .80, respectively. The protein clusters were merged with the outer membrane predictions in R to create a heatmap using the heatmap.2 from the gplots package [87]. Protein sequences of less than 60 amino acids were not included in the heat map analysis.

### RNA Isolation and Reverse Transcriptase-PCR

For RNA isolation, *B. burgdorferi* B31 cells (3 × 10^9^) were pelleted at 5,800 x *g* for 20 min at 4 °C, and the pellet was resuspended in TRI Reagent (Sigma; St. Louis, MO) before isolation of RNA according to the manufacturer’s instructions. The final RNA pellet was resuspended in 30 μl of RNase free water and was then DNase treated using the DNase I amplification grade kit from Sigma. cDNA was generated using the Phusion RT-PCR Kit (Thermo Fisher Scientific Inc, Waltham, MA) as well as specific primers listed in Table 3. For cDNA synthesis of the *uvrB*, *uvrA*, and *bb0838* operon, primers BB0838 RT 3’ and UvrA RT 3’ were used. For cDNA synthesis of the *bb0404*, *bb0405*, and *bb0406* operon, primers BB0406 RT 3’ and BB0405 RT 3’ were used. Reactions were performed in both the presence of RT (+RT) and in its absence (−RT). Subsequently, the cDNA was used for PCR analysis using PCR primer pairs listed in Table 3. In addition to cDNA, genomic DNA was included as a positive control for each reaction.

**Table 3:** Oligonucleotides utilized in the study

Primer Name	Sequence 5’-3’^*a*^	Description
BB0838 (2362) F	GCG**GCTAGC**TTATCTGATCCGGAAACTTTTTA	Cloning *bb0838* (2362) into the pET23a vector
BB0838 R	GCG**CTCGAG**TCTATTAATAATAAACTCGTAGTTT	Cloning *bb0838* (2362) into the pET23a vector
BB0405 F	GCG**GCTAGC**TCCAAAAGCAAAAGTATGACTG	Cloning *bb0405* into the pET23a vector
BB0405 R	GCG**CTCGAG**TATATATATTTTTATAAAGCCTGTG	Cloning *bb0405* into the pET23a vector
BB0406 F	GCG**GGATCC**TCTTTTGCATCTGACAATTATATG	Cloning *bb0406* into the pET23a vector
BB0406 R	GCG**CTCGAG**TGCAAATTTTATGAATCCAAATCC	Cloning *bb0406* into the pET23a vector
BB0838 RT 3’	ATCTTTAGTAAGTCCATAAGTGAAATTTT	cDNA synthesis for *uvrA*-*bb0838* PCR reaction
UvrA RT 3’	GAGCCACTCTTGCCAGATATTA	cDNA synthesis for *uvrB*-*uvrA* PCR reaction
BB0406 RT 3’	AATTCTTATAACAGCGCCTATTCTCTCATA	cDNA synthesis for *bb0405*-*bb0406* PCR reaction
BB0405 RT 3’	CATAGTTGTTCCAATAGTAGCAACAGC	cDNA synthesis for *bb0404*-*bb0405* PCR reaction
UvrB PCR 5’	GATTGTCTAAAAAAAAGCTTATTGATAAG	RT-PCR of *uvrB*-*uvrA*
UvrA PCR 3’	TGGAATATCTACATCAACATTTTTTAAATT	RT-PCR of *uvrB*-*uvrA*
UvrA PCR 5’	GTTTCTGGTATTCCTGAAGAGG	RT-PCR of *uvrA*-*bb0838*
BB0838 PCR 3’	CCAGATCCGGCAAGTCCC	RT-PCR of *uvrA*-*bb0838*
BB0404 PCR 5’	ATTAATGGCCTAAAGTTAGCTTCAAAAAG	RT-PCR of *bb0404*-*bb0405*
BB0405 PCR 3’	GCTGTACTCTATTACCAAAGGCAA	RT-PCR of *bb0404*-*bb0405*
BB0405 PCR 5’	GTTGTGATGGGTGTAGATCTTCT	RT-PCR of *bb0405*-*bb0406*
BB0406 PCR 3’	AATTCTTATAACAGCGCCTATTCTCTCATA	RT-PCR of *bb0405*-*bb0406*

^*a*^Restriction enzymes noted in bold

### Cloning, purification, and folding of candidate OMPs

Candidate OMP DNA sequences including *bb0405*, *bb0406*, and *bb0838* were amplified from *B. burgdorferi* B31 genomic DNA using primers listed in Table 3. The amplicons were subsequently digested and cloned into the NheI or BamHI and XhoI sites of pET23a (EMD Millipore, Billerica, MA). The constructs were transformed into the *E. coli* strain Rosetta 2 DE3 (EMD Millipore), and DNA sequencing was performed to verify that the sequence remained unaltered throughout the cloning process. Recombinant proteins were induced and purified using nickel-nitrilotriacetic acid agarose (Qiagen,Valencia, CA) as described previously [51]. Recombinant BB0405 and BB0406 were folded in DDM buffer [50 mM Tris, 100 mM NaCl, dodecyl-β-D-maltopyranoside (DDM; Affymetrix, 14 Santa Clara, CA)] pH 7.6 for BB0405 (0.5 % DDM) and pH 8.6 for BB0406 (2.0 % DDM) (pH and DDM concentrations were optimized for each protein) at 4 °C overnight, and the insoluble material was pelleted by centrifugation at 20,000 x *g* for 30 min at 4 °C.

### Immunoblotting and antibody production

SDS-PAGE and immunoblotting procedures were performed as described elsewhere [39, 112]. Rat polyclonal antibodies specific for BB0405 and BB0406 were generated by Harlan Bioproducts for Science, Inc. (Madison, WI) and were used at a dilution of 1:1,000 or for enhanced chemiluminescence. P66, OspA, FlaB, BB0028, Skp, OppAIV, OmpA, and GST antibodies were described previously [22, 39, 42, 51, 113]. To generate antibodies directed against BB0838, a C-terminal BB0838 peptide corresponding to the final 18 amino acids of BB0838 (E^1129^-K^1146^) was first synthesized by Thermo Fisher Scientific, and, subsequently, rabbit antisera was generated against the BB0838 C-terminal peptide (Thermo Fisher Scientific). For BB0838 immunoblots, rabbit anti-BB0838 peptide antibodies were affinity purified and used for immunoblotting at a concentration of 1:10.

### Triton X-114 phase partitioning

*B. burgdorferi* B31 whole-cell lysates were subjected to Triton X-114 phase partitioning as described elsewhere [22, 39, 114, 115] to examine the amphiphilic properties of native BB0838, BB0405, and BB0406. For BB0838, whole-cell lysates were first solubilized in 2 % DDM in PBS for two hours at room temperature before being pelleted and beginning phase partitioning which was previously described [115]. The detergent- and aqueous-enriched fractions were precipitated with acetone and subjected to SDS-PAGE and immunoblot using rabbit anti-BB0838 peptide antibody, rat anti-405, rat anti-406, rat anti-BB0028, or rat anti-Skp antibodies.

### Outer membrane preparation

*B. burgdorferi* B31 OM and protoplasmic cylinder (PC) fractions were enriched as previously described [39]. Subsequently, the fractions were separated by SDS-PAGE and immunoblotted with rabbit anti-BB0838, rat anti-BB405, or rat anti-BB406 antibodies to determine if these proteins are localized to the borrelial OM. Membranes were also immunoblotted with antibodies recognizing the known OMP P66 as well as the inner membrane lipoprotein OppAIV, which served a negative control for OM purity.

### Proteinase K and trypsin surface accessibility assays

For proteinase K accessibility experiments, 2 × 10^8^* B. burgdorferi* B31 cells were gently pelleted at 4,000 × *g* for 4 min and washed three times in PBS (pH 7.4). The final pellet was resuspended in 1 ml of PBS, and samples were aliquoted into 500 μl reactions that were either treated or mock-treated with 200 μg PK (PK; Sigma) for one hour at room temperature. Phenylmethylsulfonylfluoride (0.4 mM; Sigma) was added to each sample to stop the PK reaction, and the samples were pelleted at 10,000 × *g* for 10 min. The final pellets were prepared for SDS-PAGE and immunoblot analysis with anti-BB0838, anti-BB0405, or anti–BB0406 antibodies. Equivalent membranes were also subjected to immunoblot with antibodies to OspA or FlaB for surface and sub-surface controls, respectively. Trypsin digest assays were performed as described above for PK experiments except cells were incubated with 200 μg/ml trypsin which was resuspended in 0.001 N HCl. As controls, membranes were also immunoblotted with P66 and FlaB antibodies. Relative mobility (rf) was calculated in duplicate for full length and digested BB0838 to determine the molecular weight.

### Liposome incorporation assay

Large unilamellar vesicles (LUVs) were prepared as described [51, 115] using a mixture of 1-palmitoyl-2-oleoyl-*sn*-glycero-3-phosphocholine and 1-palmitoyl- 2-oleoyl-*sn*-glycero-3-[phospho-L-serine] (sodium salt) (Avanti Polar Lipids, Inc., Alabaster, AL), (70:30 mol %, respectively) to mimic the *B. burgdorferi* B31 OM phospholipid content [82]. For liposome incorporation assays, which were also decribed elsewhere [51, 115], recombinant BB0405 (400 ng) or BB0406 (400 ng) folded in DDM buffer were added to approximately 750 μg of LUVs in 50 mM Tris, 100 mM NaCl buffer pH 7.6 in 200 μl reactions and incubated at room temperature for 1 h. Subsequently, 200 mg of sucrose was added to each reaction, and 250 μl of 40 % sucrose followed by 300 μl of 6 % sucrose dissolved 50 mM Tris, 100 mM NaCl buffer pH 7.6 were then layered on top of the samples in ultracentrifuge tubes. The discontinuous sucrose gradients were centrifuged at 90,000 rpm for 1 h at 4 °C in a fixed angle TLA-120.2 rotor (Beckman Coulter, Brea, CA). After centrifugations, gradient fractions of equal volume were carefully collected from the top, middle, and bottom layers of the tube. Top and bottom fractions were analyzed by SDS-PAGE and immunoblot analysis with rat anti-BB0405 or rat anti-BB0406 antibodies. Control experiments were performed using recombinant *E. coli* OmpA protein folded in DDM buffer and recombinant GST protein.

### Pore formation assay

Pore formation assays were described elsewhere [116, 117]. Briefly, dried lipids were resuspended in hepes buffer containing 3 mM terbium chloride and 9 mM 2,6-pyridinedicarboxlic acid (DPA) before liposome preparation as described previously [116]. Tb(DPA)_3_^3−^ loaded liposomes were diluted in buffer containing 50 mM Tris (pH 7.5), 100 mM NaCl, and 5 mM EDTA to a concentration of 100 μM total lipids. The sample was incubated at 25 °C for 5 min, and the net initial emission intensity (*F*_0_) was determined. Next, recombinant proteins including BB0405, BB0406, P66, *E. coli* OmpA, or *E. coli* OmpF (100 nM final concentration) were added to the liposome suspension and incubated 37 °C for 30 min. Samples were then re-equilibrated to 25 °C, and the final net emission intensity (*F*_f_) of each reaction was determined after subtracting the blank and correcting for dilutions. The fraction of Tb(DPA)_3_^3−^ quenched was estimated using *F*_*f*_/*F*_0_.

## Abbreviations

OM, outer membrane; OMP, outer membrane protein; PK, proteinase K

## Additional files

Additional file 1: Table S1. Predictions for all ORFs. Lipoprotein, trans-membrane α-helix, cellular localization, and β-barrel prediction data collected for all ORFs for all genomes analyzed. (XLS 5022 kb) Additional file 2: Table S2. Candidate OMPs from various *Borrelia* genomes. List of proteins predicted to be OMPs for all genomes analyzed. (XLS 311 kb) Additional file 3: Table S3. Orthologous clusters. List of predicted orthologous clusters utilized to generate heat maps. (XLSX 667 kb) Additional file 4: Figure S1. Orthologous clusters and OMP prediction heat map. Orthologous clusters from each borrelial genome organized in unique columns as a heat map. Green blocks in each column indicate *Borrelia* proteins predicted to be OMPs, while *Borrelia* proteins not passing all OMP prediction filters are represented by red blocks. Black blocks indicate an orthologous protein was not identified in a given borrelial genome. These data were then subjected to a two-way hierarchical clustering examining orthologs (column blocks) by genomes (row blocks). Unique cluster IDs are shown at the bottom of the figure and correspond to the cluster numbers listed in Additional file 5: Table S4. Genomes analyzed are listed at right. (PDF 18 kb) Additional file 5: Table S4. Servers utilized in this study. List of all servers utilized in the study as well as URLs and references for each server. (DOCX 14 kb)
