# Supplementary material for: Consensus computational network analysis for identifying candidate outer membrane proteins from Borrelia spirochetes
Source: BMC Microbiol. 2016 Jul 11;16:141. doi: 10.1186/s12866-016-0762-z (PMC4939628; doi:10.1186/s12866-016-0762-z)
Supplement: Additional file 5: Table S4. — Servers utilized in this study. List of all servers utilized in the study as well as URLs and references for each server. (DOCX 14 kb) [file 12866_2016_762_MOESM5_ESM.docx]

**Supplementary Table 4. Servers utilized in this study.**

| Name of server | URL | Reference |
| --- | --- | --- |
| Phobius | <http://phobius.sbc.su.se/> | 56 |
| TMHMM | <http://www.cbs.dtu.dk/services/TMHMM/> | 57 |
| CELLO | <http://cello.life.nctu.edu.tw/> | 58 |
| PSORTb 3.0 | <http://www.psort.org/psortb/> | 59 |
| HHOMP | <http://toolkit.tuebingen.mpg.de/hhomp> | 60 |
| TMBETADISC-AAC | <http://rbf.bioinfo.tw/~sachen/OMPpredict/TMBETADISC-RBF.php> | 61 |
| PRED-TMBB | <http://biophysics.biol.uoa.gr/PRED-TMBB/> | 62, 111 |
| BOMP | <http://services.cbu.uib.no/tools/bomp/> | 63 |
| SignalP 3.0 | <http://www.cbs.dtu.dk/services/SignalP-3.0/> | 64 |
| PrediSi | <http://www.predisi.de/> | 65 |
| Signal-CF | <http://www.csbio.sjtu.edu.cn/bioinf/Signal-CF/> | 66 |
