# Supplementary material for: Consensus computational network analysis for identifying candidate outer membrane proteins from Borrelia spirochetes
Source: BMC Microbiol. 2016 Jul 11;16:141. doi: 10.1186/s12866-016-0762-z (PMC4939628; doi:10.1186/s12866-016-0762-z)
Supplement: Additional file 4: Figure S1. — Orthologous clusters and OMP prediction heat map. Orthologous clusters from each borrelial genome organized in unique columns as a heat map. Green blocks in each column indicate Borrelia proteins predicted to be OMPs, while Borrelia proteins not passing all OMP prediction filters are represented by red blocks. Black blocks indicate an orthologous protein was not identified in a given borrelial genome. These data were then subjected to a two-way hierarchical clustering examining orthologs (column blocks) by genomes (row blocks). Unique cluster IDs are shown at the bottom of the figure and correspond to the cluster numbers listed in Additional file 5: Table S4. Genomes analyzed are listed at right. (PDF 18 kb) [file 12866_2016_762_MOESM4_ESM.pdf]

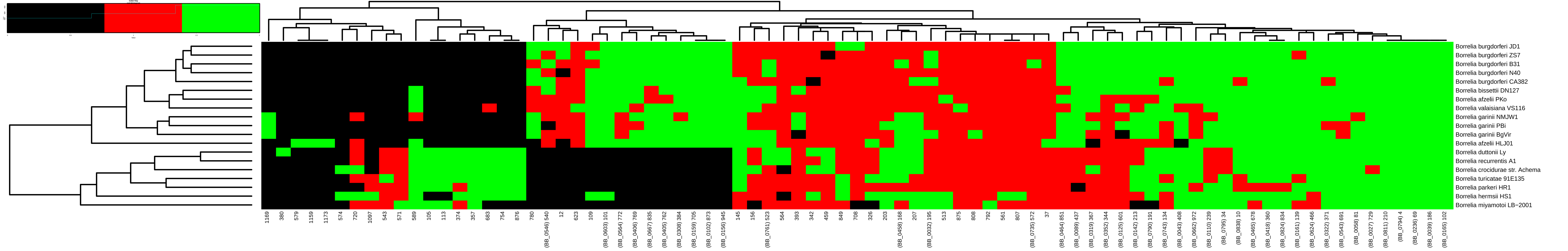

Borrelia burgdorferi JD1
Borrelia burgdorferi ZS7
Borrelia burgdorferi B31
Borrelia burgdorferi N40
Borrelia burgdorferi CA382
Borrelia bissettii DN127
Borrelia afzelii PKo
Borrelia valaisiana VS116
Borrelia garinii NMJW1
Borrelia garinii PBi
Borrelia garinii BgVir
Borrelia afzelii HLJ01
Borrelia duttonii Ly
Borrelia recurrentis A1
Borrelia crocidurae str. Achema
Borrelia turicatae 91E135
Borrelia parkeri HR1
Borrelia hermsii HS1
Borrelia miyamotoi LB−2001
1169
380
579
1159
1173
574
720
1097
543
571
589
105
113
374
357
683
754
876
780
(BB_0546) 540
12
623
109
(BB_0603) 101
(BB_0564) 772
(BB_0406) 769
(BB_0667) 835
(BB_0405) 762
(BB_0308) 384
(BB_0159) 705
(BB_0102) 873
(BB_0156) 945
145
156
(BB_0761) 523
564
393
342
459
849
708
326
203
(BB_0458) 168
207
(BB_0032) 195
513
875
808
792
561
807
(BB_0735) 572
37
(BB_0464) 851
(BB_0089) 437
(BB_0319) 367
(BB_0352) 344
(BB_0125) 601
(BB_0142) 213
(BB_0790) 191
(BB_0743) 134
(BB_0043) 408
(BB_0662) 972
(BB_0110) 239
(BB_0795) 34
(BB_0838) 10
(BB_0465) 678
(BB_0418) 360
(BB_0824) 834
(BB_0161) 139
(BB_0624) 466
(BB_0322) 371
(BB_0543) 691
(BB_0058) 81
(BB_0027) 729
(BB_0811) 210
(BB_0794) 4
(BB_0236) 69
(BB_0039) 186
(BB_0165) 102
